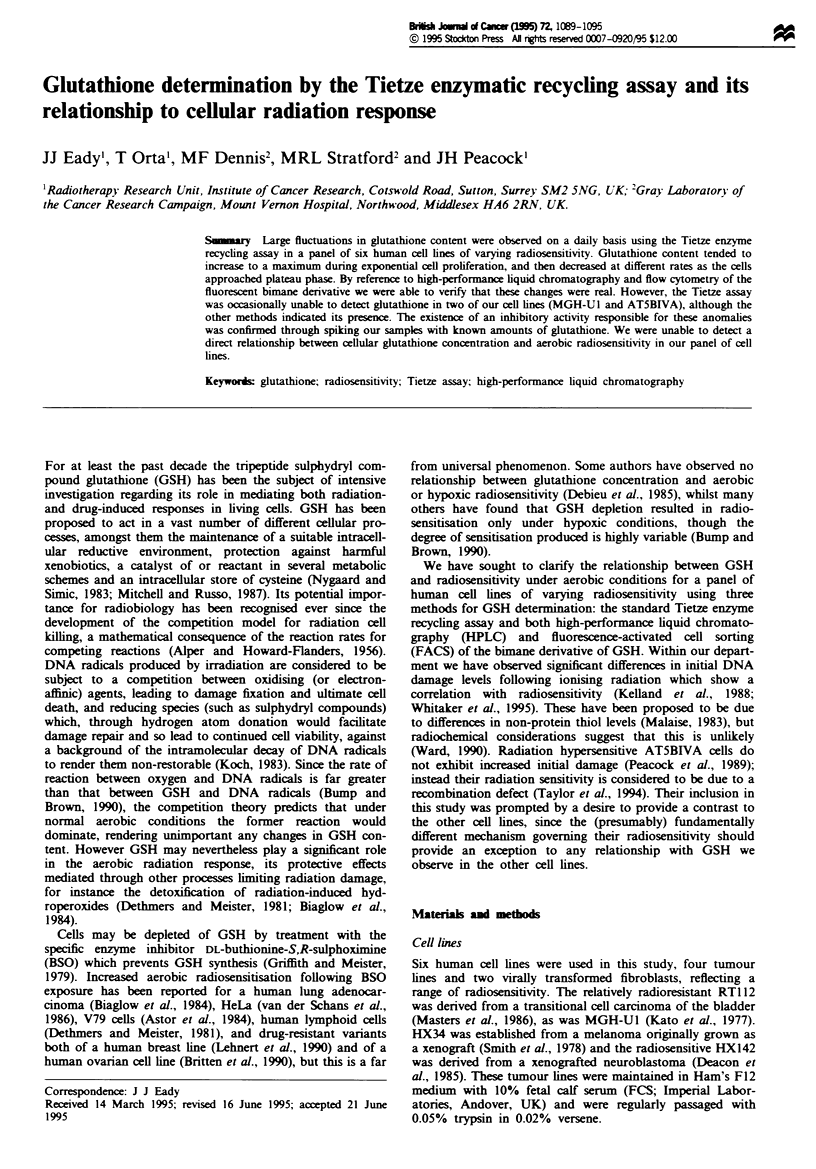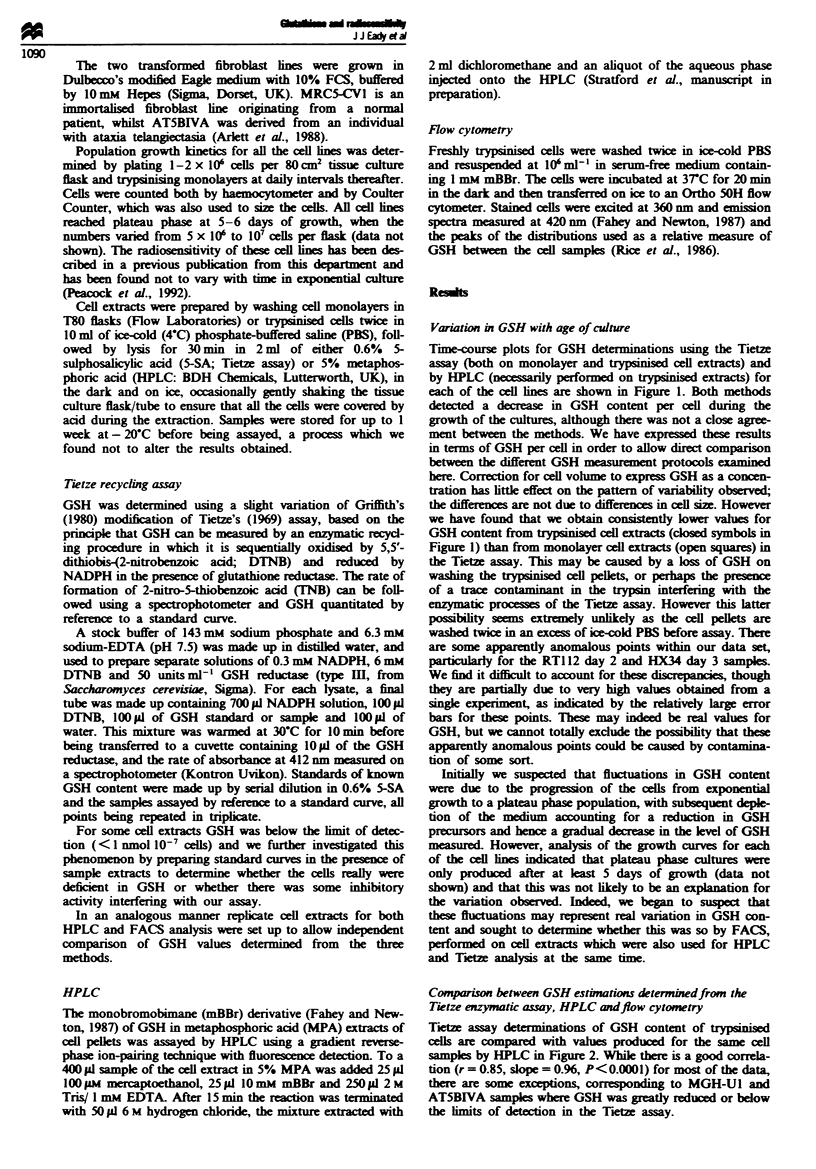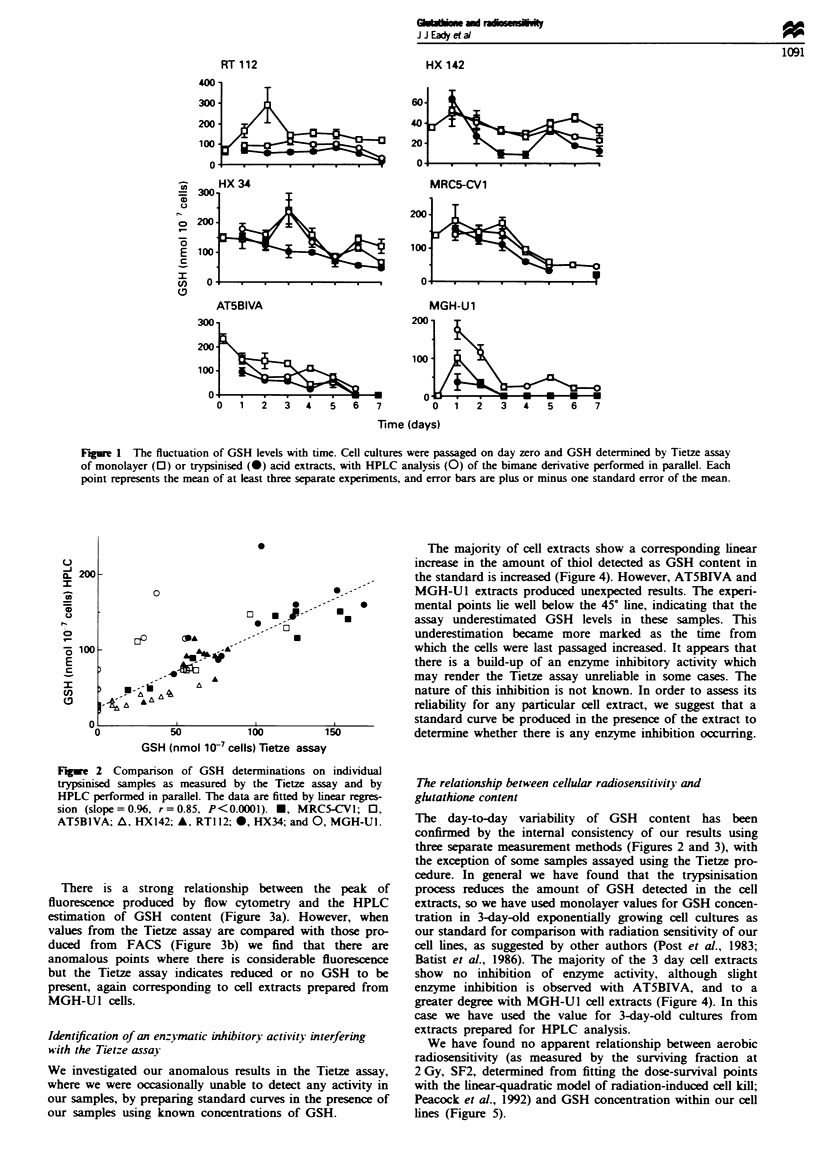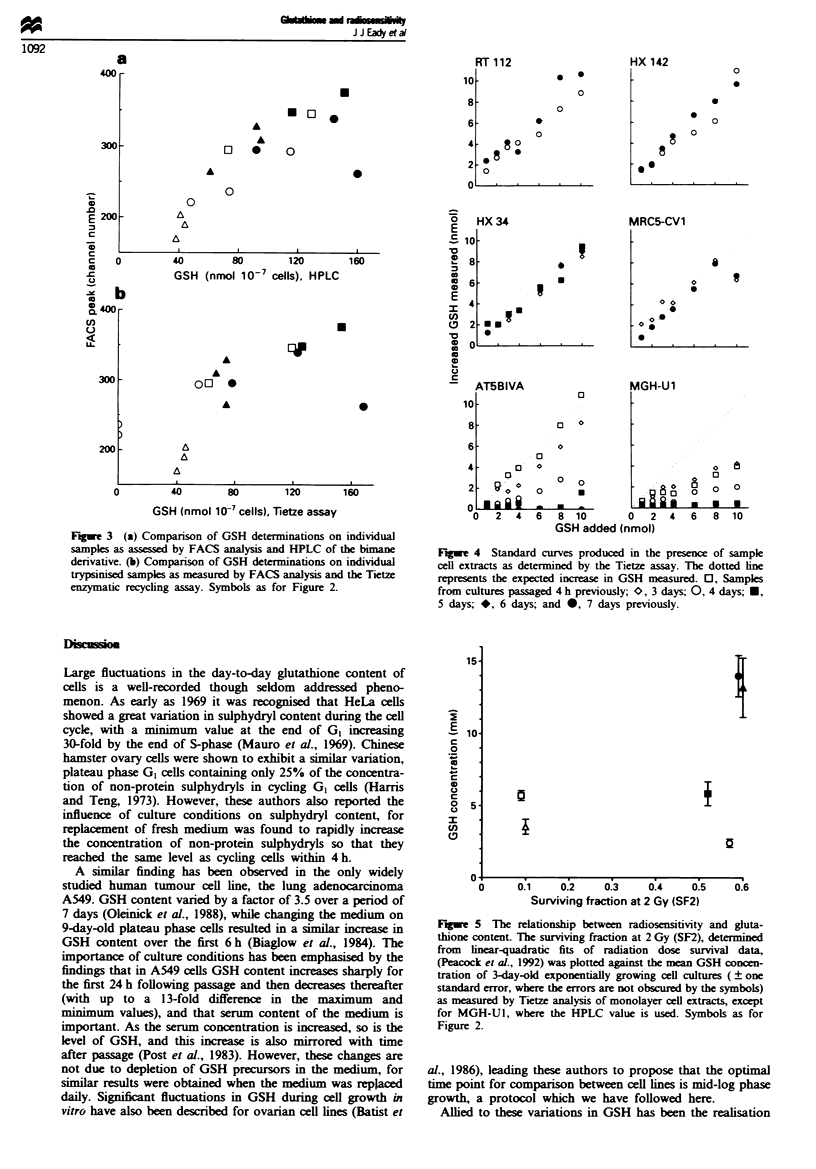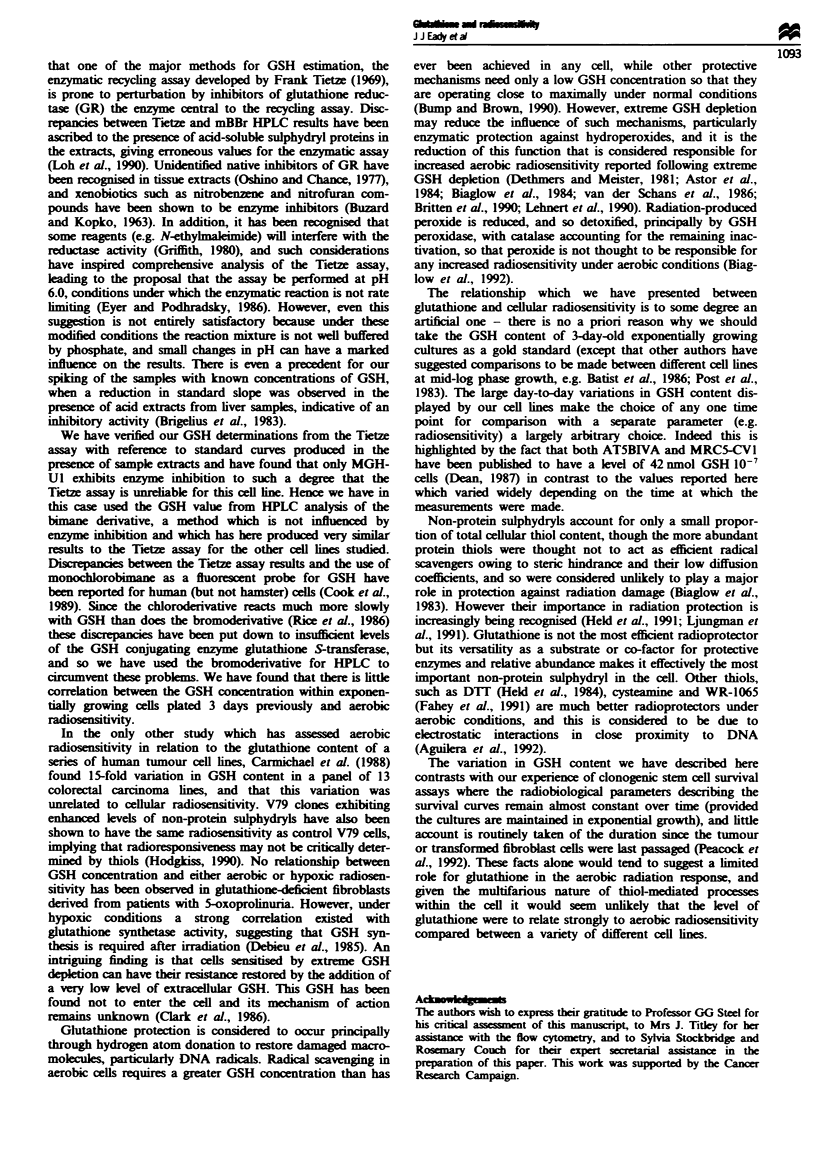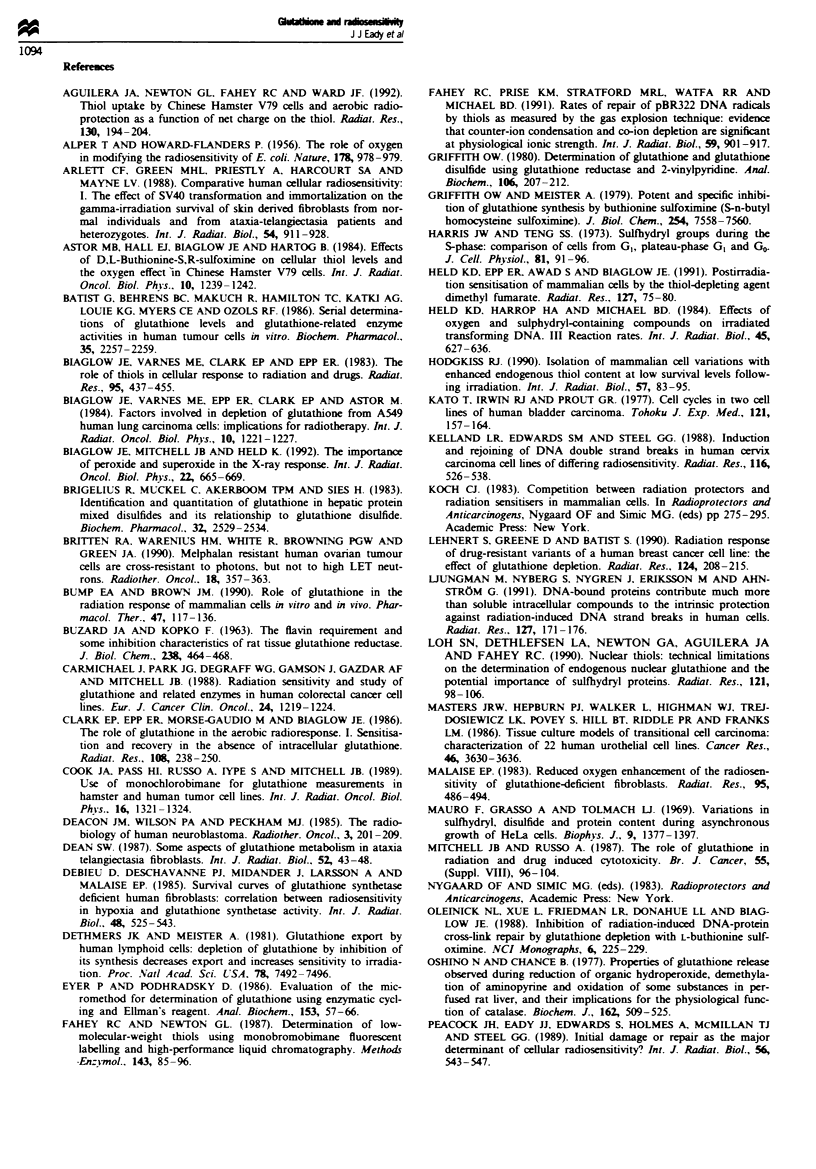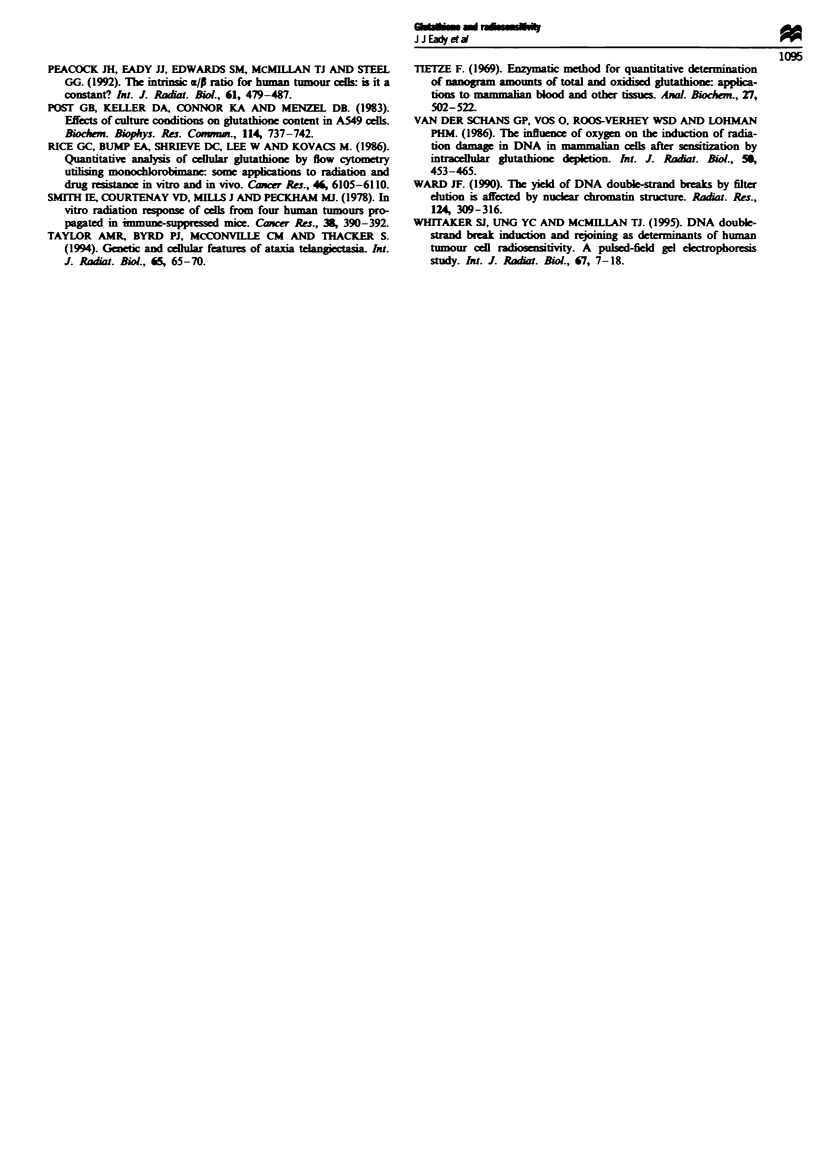# Glutathione determination by the Tietze enzymatic recycling assay and its relationship to cellular radiation response.

**DOI:** 10.1038/bjc.1995.470

**Published:** 1995-11

**Authors:** J. J. Eady, T. Orta, M. F. Dennis, M. R. Stratford, J. H. Peacock

**Affiliations:** Radiotherapy Research Unit, Institute of Cancer Research, Sutton, Surrey, UK.

## Abstract

Large fluctuations in glutathione content were observed on a daily basis using the Tietze enzyme recycling assay in a panel of six human cell lines of varying radiosensitivity. Glutathione content tended to increase to a maximum during exponential cell proliferation, and then decreased at different rates as the cells approached plateau phase. By reference to high-performance liquid chromatography and flow cytometry of the fluorescent bimane derivative we were able to verify that these changes were real. However, the Tietze assay was occasionally unable to detect glutathione in two of our cell lines (MGH-U1 and AT5BIVA), although the other methods indicated its presence. The existence of an inhibitory activity responsible for these anomalies was confirmed through spiking our samples with known amounts of glutathione. We were unable to detect a direct relationship between cellular glutathione concentration and aerobic radiosensitivity in our panel of cell lines.


British Journal of Cancer (1995) 72, 1089-1095

© 1995 Stockton Press All rights reserved 0007-0920/95 $12.00

### Glutathione determination by the Tietze enzymatic recycling assay and its relationship to cellular radiation response

JJ Eady[1], T Orta[1], MF Dennis[2], MRL Stratford[2] and JH Peacock[1]

[1]*Radiotherapy Research Unit, Institute of Cancer Research, Cotswold Road, Sutton, Surrey SM2 5NG, UK;* [2]*Gray Laboratory of the Cancer Research Campaign, Mount Vernon Hospital, Northwood, Middlesex HA6 2RN, UK.*

**Summary** Large fluctuations in glutathione content were observed on a daily basis using the Tietze enzyme recycling assay in a panel of six human cell lines of varying radiosensitivity. Glutathione content tended to increase to a maximum during exponential cell proliferation, and then decreased at different rates as the cells approached plateau phase. By reference to high-performance liquid chromatography and flow cytometry of the fluorescent bimane derivative we were able to verify that these changes were real. However, the Tietze assay was occasionally unable to detect glutathione in two of our cell lines (MGH-U1 and AT5BIVA), although the other methods indicated its presence. The existence of an inhibitory activity responsible for these anomalies was confirmed through spiking our samples with known amounts of glutathione. We were unable to detect a direct relationship between cellular glutathione concentration and aerobic radiosensitivity in our panel of cell lines.

**Keywords:** glutathione; radiosensitivity; Tietze assay; high-performance liquid chromatography

For at least the past decade the tripeptide sulphydryl compound glutathione (GSH) has been the subject of intensive investigation regarding its role in mediating both radiation- and drug-induced responses in living cells. GSH has been proposed to act in a vast number of different cellular processes, amongst them the maintenance of a suitable intracellular reductive environment, protection against harmful xenobiotics, a catalyst of or reactant in several metabolic schemes and an intracellular store of cysteine (Nygaard and Simic, 1983; Mitchell and Russo, 1987). Its potential importance for radiobiology has been recognised ever since the development of the competition model for radiation cell killing, a mathematical consequence of the reaction rates for competing reactions (Alper and Howard-Flanders, 1956). DNA radicals produced by irradiation are considered to be subject to a competition between oxidising (or electron-affinic) agents, leading to damage fixation and ultimate cell death, and reducing species (such as sulphydryl compounds) which, through hydrogen atom donation would facilitate damage repair and so lead to continued cell viability, against a background of the intramolecular decay of DNA radicals to render them non-restorable (Koch, 1983). Since the rate of reaction between oxygen and DNA radicals is far greater than that between GSH and DNA radicals (Bump and Brown, 1990), the competition theory predicts that under normal aerobic conditions the former reaction would dominate, rendering unimportant any changes in GSH content. However GSH may nevertheless play a significant role in the aerobic radiation response, its protective effects mediated through other processes limiting radiation damage, for instance the detoxification of radiation-induced hydroperoxides (Dethmers and Meister, 1981; Biaglow *et al.*, 1984).

Cells may be depleted of GSH by treatment with the specific enzyme inhibitor DL-buthionine-*S*,*R*-sulphoximine (BSO) which prevents GSH synthesis (Griffith and Meister, 1979). Increased aerobic radiosensitisation following BSO exposure has been reported for a human lung adenocarcinoma (Biaglow *et al.*, 1984), HeLa (van der Schans *et al.*, 1986), V79 cells (Astor *et al.*, 1984), human lymphoid cells (Dethmers and Meister, 1981), and drug-resistant variants both of a human breast line (Lehnert *et al.*, 1990) and of a human ovarian cell line (Britten *et al.*, 1990), but this is a far from universal phenomenon. Some authors have observed no relationship between glutathione concentration and aerobic or hypoxic radiosensitivity (Debieu *et al.*, 1985), whilst many others have found that GSH depletion resulted in radiosensitisation only under hypoxic conditions, though the degree of sensitisation produced is highly variable (Bump and Brown, 1990).

We have sought to clarify the relationship between GSH and radiosensitivity under aerobic conditions for a panel of human cell lines of varying radiosensitivity using three methods for GSH determination: the standard Tietze enzyme recycling assay and both high-performance liquid chromatography (HPLC) and fluorescence-activated cell sorting (FACS) of the bimane derivative of GSH. Within our department we have observed significant differences in initial DNA damage levels following ionising radiation which show a correlation with radiosensitivity (Kelland *et al.*, 1988; Whitaker *et al.*, 1995). These have been proposed to be due to differences in non-protein thiol levels (Malaise, 1983), but radiochemical considerations suggest that this is unlikely (Ward, 1990). Radiation hypersensitive AT5BIVA cells do not exhibit increased initial damage (Peacock *et al.*, 1989); instead their radiation sensitivity is considered to be due to a recombination defect (Taylor *et al.*, 1994). Their inclusion in this study was prompted by a desire to provide a contrast to the other cell lines, since the (presumably) fundamentally different mechanism governing their radiosensitivity should provide an exception to any relationship with GSH we observe in the other cell lines.

### Materials and methods

### *Cell lines*

Six human cell lines were used in this study, four tumour lines and two virally transformed fibroblasts, reflecting a range of radiosensitivity. The relatively radioresistant RT112 was derived from a transitional cell carcinoma of the bladder (Masters *et al.*, 1986), as was MGH-U1 (Kato *et al.*, 1977). HX34 was established from a melanoma originally grown as a xenograft (Smith *et al.*, 1978) and the radiosensitive HX142 was derived from a xenografted neuroblastoma (Deacon *et al.*, 1985). These tumour lines were maintained in Ham's F12 medium with 10% fetal calf serum (FCS; Imperial Laboratories, Andover, UK) and were regularly passaged with 0.05% trypsin in 0.02% versene.

Correspondence: J J Eady
Received 14 March 1995; revised 16 June 1995; accepted 21 June 1995

The two transformed fibroblast lines were grown in Dulbecco's modified Eagle medium with 10% FCS, buffered by 10 mM Hepes (Sigma, Dorset, UK). MRC5-CV1 is an immortalised fibroblast line originating from a normal patient, whilst AT5BIVA was derived from an individual with ataxia telangiectasia (Arlett *et al.*, 1988).

Population growth kinetics for all the cell lines was determined by plating $1-2 \times 10^6$ cells per 80 $cm^2$ tissue culture flask and trypsinising monolayers at daily intervals thereafter. Cells were counted both by haemocytometer and by Coulter Counter, which was also used to size the cells. All cell lines reached plateau phase at 5–6 days of growth, when the numbers varied from $5 \times 10^6$ to $10^7$ cells per flask (data not shown). The radiosensitivity of these cell lines has been described in a previous publication from this department and has been found not to vary with time in exponential culture (Peacock *et al.*, 1992).

Cell extracts were prepared by washing cell monolayers in T80 flasks (Flow Laboratories) or trypsinised cells twice in 10 ml of ice-cold (4°C) phosphate-buffered saline (PBS), followed by lysis for 30 min in 2 ml of either 0.6% 5-sulphosalicylic acid (5-SA; Tietze assay) or 5% metaphosphoric acid (HPLC: BDH Chemicals, Lutterworth, UK), in the dark and on ice, occasionally gently shaking the tissue culture flask/tube to ensure that all the cells were covered by acid during the extraction. Samples were stored for up to 1 week at − 20°C before being assayed, a process which we found not to alter the results obtained.

### *Tietze recycling assay*

GSH was determined using a slight variation of Griffith's (1980) modification of Tietze's (1969) assay, based on the principle that GSH can be measured by an enzymatic recycling procedure in which it is sequentially oxidised by 5,5′-dithiobis-(2-nitrobenzoic acid; DTNB) and reduced by NADPH in the presence of glutathione reductase. The rate of formation of 2-nitro-5-thiobenzoic acid (TNB) can be followed using a spectrophotometer and GSH quantitated by reference to a standard curve.

A stock buffer of 143 mM sodium phosphate and 6.3 mM sodium-EDTA (pH 7.5) was made up in distilled water, and used to prepare separate solutions of 0.3 mM NADPH, 6 mM DTNB and 50 units $ml^{-1}$ GSH reductase (type III, from *Saccharomyces cerevisiae*, Sigma). For each lysate, a final tube was made up containing 700 µl NADPH solution, 100 µl DTNB, 100 µl of GSH standard or sample and 100 µl of water. This mixture was warmed at 30°C for 10 min before being transferred to a cuvette containing 10 µl of the GSH reductase, and the rate of absorbance at 412 nm measured on a spectrophotometer (Kontron Uvikon). Standards of known GSH content were made up by serial dilution in 0.6% 5-SA and the samples assayed by reference to a standard curve, all points being repeated in triplicate.

For some cell extracts GSH was below the limit of detection ($<1$ nmol $10^{-7}$ cells) and we further investigated this phenomenon by preparing standard curves in the presence of sample extracts to determine whether the cells really were deficient in GSH or whether there was some inhibitory activity interfering with our assay.

In an analogous manner replicate cell extracts for both HPLC and FACS analysis were set up to allow independent comparison of GSH values determined from the three methods.

### *HPLC*

The monobromobimane (mBBr) derivative (Fahey and Newton, 1987) of GSH in metaphosphoric acid (MPA) extracts of cell pellets was assayed by HPLC using a gradient reverse-phase ion-pairing technique with fluorescence detection. To a 400 µl sample of the cell extract in 5% MPA was added 25 µl 100 µM mercaptoethanol, 25 µl 10 mM mBBr and 250 µl 2 M Tris/ 1 mM EDTA. After 15 min the reaction was terminated with 50 µl 6 M hydrogen chloride, the mixture extracted with 2 ml dichloromethane and an aliquot of the aqueous phase injected onto the HPLC (Stratford *et al.*, manuscript in preparation).

### *Flow cytometry*

Freshly trypsinised cells were washed twice in ice-cold PBS and resuspended at $10^6$ $ml^{-1}$ in serum-free medium containing 1 mM mBBr. The cells were incubated at 37°C for 20 min in the dark and then transferred on ice to an Ortho 50H flow cytometer. Stained cells were excited at 360 nm and emission spectra measured at 420 nm (Fahey and Newton, 1987) and the peaks of the distributions used as a relative measure of GSH between the cell samples (Rice *et al.*, 1986).

### Results

### *Variation in GSH with age of culture*

Time-course plots for GSH determinations using the Tietze assay (both on monolayer and trypsinised cell extracts) and by HPLC (necessarily performed on trypsinised extracts) for each of the cell lines are shown in Figure 1. Both methods detected a decrease in GSH content per cell during the growth of the cultures, although there was not a close agreement between the methods. We have expressed these results in terms of GSH per cell in order to allow direct comparison between the different GSH measurement protocols examined here. Correction for cell volume to express GSH as a concentration has little effect on the pattern of variability observed; the differences are not due to differences in cell size. However we have found that we obtain consistently lower values for GSH content from trypsinised cell extracts (closed symbols in Figure 1) than from monolayer cell extracts (open squares) in the Tietze assay. This may be caused by a loss of GSH on washing the trypsinised cell pellets, or perhaps the presence of a trace contaminant in the trypsin interfering with the enzymatic processes of the Tietze assay. However this latter possibility seems extremely unlikely as the cell pellets are washed twice in an excess of ice-cold PBS before assay. There are some apparently anomalous points within our data set, particularly for the RT112 day 2 and HX34 day 3 samples. We find it difficult to account for these discrepancies, though they are partially due to very high values obtained from a single experiment, as indicated by the relatively large error bars for these points. These may indeed be real values for GSH, but we cannot totally exclude the possibility that these apparently anomalous points could be caused by contamination of some sort.

Initially we suspected that fluctuations in GSH content were due to the progression of the cells from exponential growth to a plateau phase population, with subsequent depletion of the medium accounting for a reduction in GSH precursors and hence a gradual decrease in the level of GSH measured. However, analysis of the growth curves for each of the cell lines indicated that plateau phase cultures were only produced after at least 5 days of growth (data not shown) and that this was not likely to be an explanation for the variation observed. Indeed, we began to suspect that these fluctuations may represent real variation in GSH content and sought to determine whether this was so by FACS, performed on cell extracts which were also used for HPLC and Tietze analysis at the same time.

### *Comparison between GSH estimations determined from the Tietze enzymatic assay, HPLC and flow cytometry*

Tietze assay determinations of GSH content of trypsinised cells are compared with values produced for the same cell samples by HPLC in Figure 2. While there is a good correlation ($r = 0.85$, slope = 0.96, $P < 0.0001$) for most of the data, there are some exceptions, corresponding to MGH-U1 and AT5BIVA samples where GSH was greatly reduced or below the limits of detection in the Tietze assay.

**Figure 1** The fluctuation of GSH levels with time. Cell cultures were passaged on day zero and GSH determined by Tietze assay of monolayer (□) or trypsinised (●) acid extracts, with HPLC analysis (O) of the bimane derivative performed in parallel. Each point represents the mean of at least three separate experiments, and error bars are plus or minus one standard error of the mean.

**Figure 2** Comparison of GSH determinations on individual trypsinised samples as measured by the Tietze assay and by HPLC performed in parallel. The data are fitted by linear regression (slope = 0.96, $r = 0.85$, $P < 0.0001$). ■, MRC5-CV1; □, AT5B1VA; Δ, HX142; ▲, RT112; ●, HX34; and O, MGH-U1.

There is a strong relationship between the peak of fluorescence produced by flow cytometry and the HPLC estimation of GSH content (Figure 3a). However, when values from the Tietze assay are compared with those produced from FACS (Figure 3b) we find that there are anomalous points where there is considerable fluorescence but the Tietze assay indicates reduced or no GSH to be present, again corresponding to cell extracts prepared from MGH-U1 cells.

*Identification of an enzymatic inhibitory activity interfering with the Tietze assay*

We investigated our anomalous results in the Tietze assay, where we were occasionally unable to detect any activity in our samples, by preparing standard curves in the presence of our samples using known concentrations of GSH.

The majority of cell extracts show a corresponding linear increase in the amount of thiol detected as GSH content in the standard is increased (Figure 4). However, AT5BIVA and MGH-U1 extracts produced unexpected results. The experimental points lie well below the 45° line, indicating that the assay underestimated GSH levels in these samples. This underestimation became more marked as the time from which the cells were last passaged increased. It appears that there is a build-up of an enzyme inhibitory activity which may render the Tietze assay unreliable in some cases. The nature of this inhibition is not known. In order to assess its reliability for any particular cell extract, we suggest that a standard curve be produced in the presence of the extract to determine whether there is any enzyme inhibition occurring.

*The relationship between cellular radiosensitivity and glutathione content*

The day-to-day variability of GSH content has been confirmed by the internal consistency of our results using three separate measurement methods (Figures 2 and 3), with the exception of some samples assayed using the Tietze procedure. In general we have found that the trypsinisation process reduces the amount of GSH detected in the cell extracts, so we have used monolayer values for GSH concentration in 3-day-old exponentially growing cell cultures as our standard for comparison with radiation sensitivity of our cell lines, as suggested by other authors (Post *et al.*, 1983; Batist *et al.*, 1986). The majority of the 3 day cell extracts show no inhibition of enzyme activity, although slight enzyme inhibition is observed with AT5BIVA, and to a greater degree with MGH-U1 cell extracts (Figure 4). In this case we have used the value for 3-day-old cultures from extracts prepared for HPLC analysis.

We have found no apparent relationship between aerobic radiosensitivity (as measured by the surviving fraction at 2 Gy, SF2, determined from fitting the dose-survival points with the linear-quadratic model of radiation-induced cell kill; Peacock *et al.*, 1992) and GSH concentration within our cell lines (Figure 5).

**Figure 3** (**a**) Comparison of GSH determinations on individual samples as assessed by FACS analysis and HPLC of the bimane derivative. (**b**) Comparison of GSH determinations on individual trypsinised samples as measured by FACS analysis and the Tietze enzymatic recycling assay. Symbols as for Figure 2.

**Figure 4** Standard curves produced in the presence of sample cell extracts as determined by the Tietze assay. The dotted line represents the expected increase in GSH measured. □, Samples from cultures passaged 4 h previously; ◇, 3 days; ○, 4 days; ■, 5 days; ◆, 6 days; and ●, 7 days previously.

### Discussion

Large fluctuations in the day-to-day glutathione content of cells is a well-recorded though seldom addressed phenomenon. As early as 1969 it was recognised that HeLa cells showed a great variation in sulphydryl content during the cell cycle, with a minimum value at the end of $G_1$ increasing 30-fold by the end of S-phase (Mauro *et al.*, 1969). Chinese hamster ovary cells were shown to exhibit a similar variation, plateau phase $G_1$ cells containing only 25% of the concentration of non-protein sulphydryls in cycling $G_1$ cells (Harris and Teng, 1973). However, these authors also reported the influence of culture conditions on sulphydryl content, for replacement of fresh medium was found to rapidly increase the concentration of non-protein sulphydryls so that they reached the same level as cycling cells within 4 h.

A similar finding has been observed in the only widely studied human tumour cell line, the lung adenocarcinoma A549. GSH content varied by a factor of 3.5 over a period of 7 days (Oleinick *et al.*, 1988), while changing the medium on 9-day-old plateau phase cells resulted in a similar increase in GSH content over the first 6 h (Biaglow *et al.*, 1984). The importance of culture conditions has been emphasised by the findings that in A549 cells GSH content increases sharply for the first 24 h following passage and then decreases thereafter (with up to a 13-fold difference in the maximum and minimum values), and that serum content of the medium is important. As the serum concentration is increased, so is the level of GSH, and this increase is also mirrored with time after passage (Post *et al.*, 1983). However, these changes are not due to depletion of GSH precursors in the medium, for similar results were obtained when the medium was replaced daily. Significant fluctuations in GSH during cell growth *in vitro* have also been described for ovarian cell lines (Batist *et al.*, 1986), leading these authors to propose that the optimal time point for comparison between cell lines is mid-log phase growth, a protocol which we have followed here.

**Figure 5** The relationship between radiosensitivity and glutathione content. The surviving fraction at 2 Gy (SF2), determined from linear-quadratic fits of radiation dose survival data, (Peacock *et al.*, 1992) was plotted against the mean GSH concentration of 3-day-old exponentially growing cell cultures (± one standard error, where the errors are not obscured by the symbols) as measured by Tietze analysis of monolayer cell extracts, except for MGH-U1, where the HPLC value is used. Symbols as for Figure 2.

Allied to these variations in GSH has been the realisation that one of the major methods for GSH estimation, the enzymatic recycling assay developed by Frank Tietze (1969), is prone to perturbation by inhibitors of glutathione reductase (GR) the enzyme central to the recycling assay. Discrepancies between Tietze and mBBr HPLC results have been ascribed to the presence of acid-soluble sulphydryl proteins in the extracts, giving erroneous values for the enzymatic assay (Loh *et al.*, 1990). Unidentified native inhibitors of GR have been recognised in tissue extracts (Oshino and Chance, 1977), and xenobiotics such as nitrobenzene and nitrofuran compounds have been shown to be enzyme inhibitors (Buzard and Kopko, 1963). In addition, it has been recognised that some reagents (e.g. *N*-ethylmaleimide) will interfere with the reductase activity (Griffith, 1980), and such considerations have inspired comprehensive analysis of the Tietze assay, leading to the proposal that the assay be performed at pH 6.0, conditions under which the enzymatic reaction is not rate limiting (Eyer and Podhradsky, 1986). However, even this suggestion is not entirely satisfactory because under these modified conditions the reaction mixture is not well buffered by phosphate, and small changes in pH can have a marked influence on the results. There is even a precedent for our spiking of the samples with known concentrations of GSH, when a reduction in standard slope was observed in the presence of acid extracts from liver samples, indicative of an inhibitory activity (Brigelius *et al.*, 1983).

We have verified our GSH determinations from the Tietze assay with reference to standard curves produced in the presence of sample extracts and have found that only MGH-U1 exhibits enzyme inhibition to such a degree that the Tietze assay is unreliable for this cell line. Hence we have in this case used the GSH value from HPLC analysis of the bimane derivative, a method which is not influenced by enzyme inhibition and which has here produced very similar results to the Tietze assay for the other cell lines studied. Discrepancies between the Tietze assay results and the use of monochlorobimane as a fluorescent probe for GSH have been reported for human (but not hamster) cells (Cook *et al.*, 1989). Since the chloroderivative reacts much more slowly with GSH than does the bromoderivative (Rice *et al.*, 1986) these discrepancies have been put down to insufficient levels of the GSH conjugating enzyme glutathione *S*-transferase, and so we have used the bromoderivative for HPLC to circumvent these problems. We have found that there is little correlation between the GSH concentration within exponentially growing cells plated 3 days previously and aerobic radiosensitivity.

In the only other study which has assessed aerobic radiosensitivity in relation to the glutathione content of a series of human tumour cell lines, Carmichael *et al.* (1988) found 15-fold variation in GSH content in a panel of 13 colorectal carcinoma lines, and that this variation was unrelated to cellular radiosensitivity. V79 clones exhibiting enhanced levels of non-protein sulphydryls have also been shown to have the same radiosensitivity as control V79 cells, implying that radioresponsiveness may not be critically determined by thiols (Hodgkiss, 1990). No relationship between GSH concentration and either aerobic or hypoxic radiosensitivity has been observed in glutathione-deficient fibroblasts derived from patients with 5-oxoprolinuria. However, under hypoxic conditions a strong correlation existed with glutathione synthetase activity, suggesting that GSH synthesis is required after irradiation (Debieu *et al.*, 1985). An intriguing finding is that cells sensitised by extreme GSH depletion can have their resistance restored by the addition of a very low level of extracellular GSH. This GSH has been found not to enter the cell and its mechanism of action remains unknown (Clark *et al.*, 1986).

Glutathione protection is considered to occur principally through hydrogen atom donation to restore damaged macromolecules, particularly DNA radicals. Radical scavenging in aerobic cells requires a greater GSH concentration than has ever been achieved in any cell, while other protective mechanisms need only a low GSH concentration so that they are operating close to maximally under normal conditions (Bump and Brown, 1990). However, extreme GSH depletion may reduce the influence of such mechanisms, particularly enzymatic protection against hydroperoxides, and it is the reduction of this function that is considered responsible for increased aerobic radiosensitivity reported following extreme GSH depletion (Dethmers and Meister, 1981; Astor *et al.*, 1984; Biaglow *et al.*, 1984; van der Schans *et al.*, 1986; Britten *et al.*, 1990; Lehnert *et al.*, 1990). Radiation-produced peroxide is reduced, and so detoxified, principally by GSH peroxidase, with catalase accounting for the remaining inactivation, so that peroxide is not thought to be responsible for any increased radiosensitivity under aerobic conditions (Biaglow *et al.*, 1992).

The relationship which we have presented between glutathione and cellular radiosensitivity is to some degree an artificial one – there is no a priori reason why we should take the GSH content of 3-day-old exponentially growing cultures as a gold standard (except that other authors have suggested comparisons to be made between different cell lines at mid-log phase growth, e.g. Batist *et al.*, 1986; Post *et al.*, 1983). The large day-to-day variations in GSH content displayed by our cell lines make the choice of any one time point for comparison with a separate parameter (e.g. radiosensitivity) a largely arbitrary choice. Indeed this is highlighted by the fact that both AT5BIVA and MRC5-CV1 have been published to have a level of 42 nmol GSH $10^{-7}$ cells (Dean, 1987) in contrast to the values reported here which varied widely depending on the time at which the measurements were made.

Non-protein sulphydryls account for only a small proportion of total cellular thiol content, though the more abundant protein thiols were thought not to act as efficient radical scavengers owing to steric hindrance and their low diffusion coefficients, and so were considered unlikely to play a major role in protection against radiation damage (Biaglow *et al.*, 1983). However their importance in radiation protection is increasingly being recognised (Held *et al.*, 1991; Ljungman *et al.*, 1991). Glutathione is not the most efficient radioprotector but its versatility as a substrate or co-factor for protective enzymes and relative abundance makes it effectively the most important non-protein sulphydryl in the cell. Other thiols, such as DTT (Held *et al.*, 1984), cysteamine and WR-1065 (Fahey *et al.*, 1991) are much better radioprotectors under aerobic conditions, and this is considered to be due to electrostatic interactions in close proximity to DNA (Aguilera *et al.*, 1992).

The variation in GSH content we have described here contrasts with our experience of clonogenic stem cell survival assays where the radiobiological parameters describing the survival curves remain almost constant over time (provided the cultures are maintained in exponential growth), and little account is routinely taken of the duration since the tumour or transformed fibroblast cells were last passaged (Peacock *et al.*, 1992). These facts alone would tend to suggest a limited role for glutathione in the aerobic radiation response, and given the multifarious nature of thiol-mediated processes within the cell it would seem unlikely that the level of glutathione were to relate strongly to aerobic radiosensitivity compared between a variety of different cell lines.

**Acknowledgements**

The authors wish to express their gratitude to Professor GG Steel for his critical assessment of this manuscript, to Mrs J. Titley for her assistance with the flow cytometry, and to Sylvia Stockbridge and Rosemary Couch for their expert secretarial assistance in the preparation of this paper. This work was supported by the Cancer Research Campaign.

**References**

AGUILERA JA, NEWTON GL, FAHEY RC AND WARD JF. (1992). Thiol uptake by Chinese Hamster V79 cells and aerobic radioprotection as a function of net charge on the thiol. *Radiat. Res.*, **130**, 194–204.

ALPER T AND HOWARD-FLANDERS P. (1956). The role of oxygen in modifying the radiosensitivity of *E. coli*. *Nature*, **178**, 978–979.

ARLETT CF, GREEN MHL, PRIESTLY A, HARCOURT SA AND MAYNE LV. (1988). Comparative human cellular radiosensitivity: I. The effect of SV40 transformation and immortalization on the gamma-irradiation survival of skin derived fibroblasts from normal individuals and from ataxia-telangiectasia patients and heterozygotes. *Int. J. Radiat. Biol.*, **54**, 911–928.

ASTOR MB, HALL EJ, BIAGLOW JE AND HARTOG B. (1984). Effects of D,L-Buthionine-S,R-sulfoximine on cellular thiol levels and the oxygen effect in Chinese Hamster V79 cells. *Int. J. Radiat. Oncol. Biol. Phys.*, **10**, 1239–1242.

BATIST G, BEHRENS BC, MAKUCH R, HAMILTON TC, KATKI AG, LOUIE KG, MYERS CE AND OZOLS RF. (1986). Serial determinations of glutathione levels and glutathione-related enzyme activities in human tumour cells *in vitro*. *Biochem. Pharmacol.*, **35**, 2257–2259.

BIAGLOW JE, VARNES ME, CLARK EP AND EPP ER. (1983). The role of thiols in cellular response to radiation and drugs. *Radiat. Res.*, **95**, 437–455.

BIAGLOW JE, VARNES ME, EPP ER, CLARK EP AND ASTOR M. (1984). Factors involved in depletion of glutathione from A549 human lung carcinoma cells: implications for radiotherapy. *Int. J. Radiat. Oncol. Biol. Phys.*, **10**, 1221–1227.

BIAGLOW JE, MITCHELL JB AND HELD K. (1992). The importance of peroxide and superoxide in the X-ray response. *Int. J. Radiat. Oncol. Biol. Phys.*, **22**, 665–669.

BRIGELIUS R, MUCKEL C, AKERBOOM TPM AND SIES H. (1983). Identification and quantitation of glutathione in hepatic protein mixed disulfides and its relationship to glutathione disulfide. *Biochem. Pharmacol.*, **32**, 2529–2534.

BRITTEN RA, WARENIUS HM, WHITE R, BROWNING PGW AND GREEN JA. (1990). Melphalan resistant human ovarian tumour cells are cross-resistant to photons, but not to high LET neutrons. *Radiother. Oncol.*, **18**, 357–363.

BUMP EA AND BROWN JM. (1990). Role of glutathione in the radiation response of mammalian cells *in vitro* and *in vivo*. *Pharmacol. Ther.*, **47**, 117–136.

BUZARD JA AND KOPKO F. (1963). The flavin requirement and some inhibition characteristics of rat tissue glutathione reductase. *J. Biol. Chem.*, **238**, 464–468.

CARMICHAEL J, PARK JG, DEGRAFF WG, GAMSON J, GAZDAR AF AND MITCHELL JB. (1988). Radiation sensitivity and study of glutathione and related enzymes in human colorectal cancer cell lines. *Eur. J. Cancer Clin. Oncol.*, **24**, 1219–1224.

CLARK EP, EPP ER, MORSE-GAUDIO M AND BIAGLOW JE. (1986). The role of glutathione in the aerobic radioresponse. I. Sensitisation and recovery in the absence of intracellular glutathione. *Radiat. Res.*, **108**, 238–250.

COOK JA, PASS HI, RUSSO A, IYPE S AND MITCHELL JB. (1989). Use of monochlorobimane for glutathione measurements in hamster and human tumor cell lines. *Int. J. Radiat. Oncol. Biol. Phys.*, **16**, 1321–1324.

DEACON JM, WILSON PA AND PECKHAM MJ. (1985). The radiobiology of human neuroblastoma. *Radiother. Oncol.*, **3**, 201–209.

DEAN SW. (1987). Some aspects of glutathione metabolism in ataxia telangiectasia fibroblasts. *Int. J. Radiat. Biol.*, **52**, 43–48.

DEBIEU D, DESCHAVANNE PJ, MIDANDER J, LARSSON A AND MALAISE EP. (1985). Survival curves of glutathione synthetase deficient human fibroblasts: correlation between radiosensitivity in hypoxia and glutathione synthetase activity. *Int. J. Radiat. Biol.*, **48**, 525–543.

DETHMERS JK AND MEISTER A. (1981). Glutathione export by human lymphoid cells: depletion of glutathione by inhibition of its synthesis decreases export and increases sensitivity to irradiation. *Proc. Natl Acad. Sci. USA*, **78**, 7492–7496.

EYER P AND PODHRADSKY D. (1986). Evaluation of the micromethod for determination of glutathione using enzymatic cycling and Ellman's reagent. *Anal. Biochem.*, **153**, 57–66.

FAHEY RC AND NEWTON GL. (1987). Determination of low-molecular-weight thiols using monobromobimane fluorescent labelling and high-performance liquid chromatography. *Methods Enzymol.*, **143**, 85–96.

FAHEY RC, PRISE KM, STRATFORD MRL, WATFA RR AND MICHAEL BD. (1991). Rates of repair of pBR322 DNA radicals by thiols as measured by the gas explosion technique: evidence that counter-ion condensation and co-ion depletion are significant at physiological ionic strength. *Int. J. Radiat. Biol.*, **59**, 901–917.

GRIFFITH OW. (1980). Determination of glutathione and glutathione disulfide using glutathione reductase and 2-vinylpyridine. *Anal. Biochem.*, **106**, 207–212.

GRIFFITH OW AND MEISTER A. (1979). Potent and specific inhibition of glutathione synthesis by buthionine sulfoximine (S-n-butyl homocysteine sulfoximine). *J. Biol. Chem.*, **254**, 7558–7560.

HARRIS JW AND TENG SS. (1973). Sulfhydryl groups during the S-phase: comparison of cells from $G_1$, plateau-phase $G_1$ and $G_0$. *J. Cell. Physiol.*, **81**, 91–96.

HELD KD, EPP ER, AWAD S AND BIAGLOW JE. (1991). Postirradiation sensitisation of mammalian cells by the thiol-depleting agent dimethyl fumarate. *Radiat. Res.*, **127**, 75–80.

HELD KD, HARROP HA AND MICHAEL BD. (1984). Effects of oxygen and sulphydryl-containing compounds on irradiated transforming DNA. III Reaction rates. *Int. J. Radiat. Biol.*, **45**, 627–636.

HODGKISS RJ. (1990). Isolation of mammalian cell variations with enhanced endogenous thiol content at low survival levels following irradiation. *Int. J. Radiat. Biol.*, **57**, 83–95.

KATO T, IRWIN RJ AND PROUT GR. (1977). Cell cycles in two cell lines of human bladder carcinoma. *Tohoku J. Exp. Med.*, **121**, 157–164.

KELLAND LR, EDWARDS SM AND STEEL GG. (1988). Induction and rejoining of DNA double strand breaks in human cervix carcinoma cell lines of differing radiosensitivity. *Radiat. Res.*, **116**, 526–538.

KOCH CJ. (1983). Competition between radiation protectors and radiation sensitisers in mammalian cells. In *Radioprotectors and Anticarcinogens*, Nygaard OF and Simic MG. (eds) pp 275–295. Academic Press: New York.

LEHNERT S, GREENE D AND BATIST S. (1990). Radiation response of drug-resistant variants of a human breast cancer cell line: the effect of glutathione depletion. *Radiat. Res.*, **124**, 208–215.

LJUNGMAN M, NYBERG S, NYGREN J, ERIKSSON M AND AHNSTRÖM G. (1991). DNA-bound proteins contribute much more than soluble intracellular compounds to the intrinsic protection against radiation-induced DNA strand breaks in human cells. *Radiat. Res.*, **127**, 171–176.

LOH SN, DETHLEFSEN LA, NEWTON GA, AGUILERA JA AND FAHEY RC. (1990). Nuclear thiols: technical limitations on the determination of endogenous nuclear glutathione and the potential importance of sulfhydryl proteins. *Radiat. Res.*, **121**, 98–106.

MASTERS JRW, HEPBURN PJ, WALKER L, HIGHMAN WJ, TREJDOSIEWICZ LK, POVEY S, HILL BT, RIDDLE PR AND FRANKS LM. (1986). Tissue culture models of transitional cell carcinoma: characterization of 22 human urothelial cell lines. *Cancer Res.*, **46**, 3630–3636.

MALAISE EP. (1983). Reduced oxygen enhancement of the radiosensitivity of glutathione-deficient fibroblasts. *Radiat. Res.*, **95**, 486–494.

MAURO F, GRASSO A AND TOLMACH LJ. (1969). Variations in sulfhydryl, disulfide and protein content during asynchronous growth of HeLa cells. *Biophys. J.*, **9**, 1377–1397.

MITCHELL JB AND RUSSO A. (1987). The role of glutathione in radiation and drug induced cytotoxicity. *Br. J. Cancer*, **55**, (Suppl. VIII), 96–104.

NYGAARD OF AND SIMIC MG. (eds). (1983). *Radioprotectors and Anticarcinogens*, Academic Press: New York.

OLEINICK NL, XUE L, FRIEDMAN LR, DONAHUE LL AND BIAGLOW JE. (1988). Inhibition of radiation-induced DNA-protein cross-link repair by glutathione depletion with L-buthionine sulfoximine. *NCI Monographs*, **6**, 225–229.

OSHINO N AND CHANCE B. (1977). Properties of glutathione release observed during reduction of organic hydroperoxide, demethylation of aminopyrine and oxidation of some substances in perfused rat liver, and their implications for the physiological function of catalase. *Biochem. J.*, **162**, 509–525.

PEACOCK JH, EADY JJ, EDWARDS S, HOLMES A, MCMILLAN TJ AND STEEL GG. (1989). Initial damage or repair as the major determinant of cellular radiosensitivity? *Int. J. Radiat. Biol.*, **56**, 543–547.

PEACOCK JH, EADY JJ, EDWARDS SM, MCMILLAN TJ AND STEEL GG. (1992). The intrinsic $\alpha/\beta$ ratio for human tumour cells: is it a constant? *Int. J. Radiat. Biol.*, **61**, 479–487.

POST GB, KELLER DA, CONNOR KA AND MENZEL DB. (1983). Effects of culture conditions on glutathione content in A549 cells. *Biochem. Biophys. Res. Commun.*, **114**, 737–742.

RICE GC, BUMP EA, SHRIEVE DC, LEE W AND KOVACS M. (1986). Quantitative analysis of cellular glutathione by flow cytometry utilising monochlorobimane: some applications to radiation and drug resistance in vitro and in vivo. *Cancer Res.*, **46**, 6105–6110.

SMITH IE, COURTENAY VD, MILLS J AND PECKHAM MJ. (1978). In vitro radiation response of cells from four human tumours propagated in immune-suppressed mice. *Cancer Res.*, **38**, 390–392.

TAYLOR AMR, BYRD PJ, MCCONVILLE CM AND THACKER S. (1994). Genetic and cellular features of ataxia telangiectasia. *Int. J. Radiat. Biol.*, **65**, 65–70.

TIETZE F. (1969). Enzymatic method for quantitative determination of nanogram amounts of total and oxidised glutathione: applications to mammalian blood and other tissues. *Anal. Biochem.*, **27**, 502–522.

VAN DER SCHANS GP, VOS O, ROOS-VERHEY WSD AND LOHMAN PHM. (1986). The influence of oxygen on the induction of radiation damage in DNA in mammalian cells after sensitization by intracellular glutathione depletion. *Int. J. Radiat. Biol.*, **50**, 453–465.

WARD JF. (1990). The yield of DNA double-strand breaks by filter elution is affected by nuclear chromatin structure. *Radiat. Res.*, **124**, 309–316.

WHITAKER SJ, UNG YC AND MCMILLAN TJ. (1995). DNA double-strand break induction and rejoining as determinants of human tumour cell radiosensitivity. A pulsed-field gel electrophoresis study. *Int. J. Radiat. Biol.*, **67**, 7–18.